# Our July Number

**Published:** 1857-07

**Authors:** 


*Our July Number.*—At last we have nearly caught up with the times and bring out our number for July with some regard for the first of the month. The delay in the issue of recent numbers has been owing to a cause for which ourselves and our misfortunes are alone responsible. Our publishers are ever prompt, and the entire routine of the typographical execution of the Journal is in a most satisfactory condition of order and system. But we of the chair editorial, after dragging through a winter of constant labor and exertion, with more duties than time, found ourselves at its close merged in the sorrow of a domestic affliction, and sought in a brief respite from toil a necessary relief. Once behind hand it is no easy matter to bring up again, and so we have gone on in a dilatory manner. We hope now to keep and improve upon the ground we have gained.

